# Predictors of Late-Life Divorce in South Korea: A 21-Year Panel Study

**DOI:** 10.1093/geroni/igaf122.3491

**Published:** 2025-12-31

**Authors:** Seoyeon Ahn, Minyoung Kwak, Sojung Park

**Affiliations:** Duke-NUS Medical School/National University of Singapore, SINGAPORE, Singapore; Daegu University, Gyongsan, Kyongsang-bukto, Republic of Korea; Washington University in St. Louis, St. Louis, Missouri, United States

## Abstract

This study examines how socioeconomic and marital factors influence late-life separation and divorce men and women aged over 50 in Korea. Using data from the 21-wave Korea Labor and Income Panel Study (1999–2019), we analyze a sample of 4,922 married couples aged over 50 years old. Discrete-time event history models are applied to investigate the impact of socioeconomic and marital factors on marital instability over the 21-year period. Our findings indicate that home ownership and husbands’ pension receipt significantly reduce the risk of marital dissolution, while wives’ secondary education is associated with a higher likelihood of marital disruption. Additionally, Husbands’ higher levels of relationship satisfaction with family was associated with lower risk of marital disruption. These results highlight the importance of considering both socioeconomic resources and interpersonal factors in explaining marital instability among middle-aged and older adults. The complex relationship between education and divorce risk for older Korean women suggests that both financial independence and cultural norms shape divorce decisions. These findings contribute to our understanding of gray divorce in East Asian societies, where traditional family values coexist with rapid socioeconomic change. Recognizing the predictors of late-life marital dissolution has important implications for developing social services tailored to the unique needs of divorced older adults, particularly in aging societies with evolving family structures.

